# Efficacy of bosentan in patients with refractory thromboangiitis obliterans (Buerger disease): A case series and review of the literature

**DOI:** 10.1097/MD.0000000000005511

**Published:** 2016-12-02

**Authors:** Javier Narváez, Carmen García-Gómez, Lorenzo Álvarez, Pilar Santo, María Aparicio, María Pascual, Mercè López de Recalde, Helena Borrell, Joan M. Nolla

**Affiliations:** a Department of Rheumatology. Hospital Universitario de Bellvitge-IDIBELL; b Rheumatology Unit; c Department of Angiology and Vascular Surgery, Consorci Sanitari de Terrassa; d Department of Rheumatology, Parc Sanitari Sant Joan de Déu, Sant Boi, Barcelona, Spain.

**Keywords:** bosentan, Buerger disease, effectiveness, safety, thromboangiitis obliterans

## Abstract

The cornerstone of therapy in thromboangiitis obliterans (TAO) is complete abstinence from tobacco. In addition to discontinuation of cigarette smoking, very few pharmacological and surgical options of controversial efficacy are available to date. New therapeutic options with greater efficacy are clearly needed to properly manage these patients.

In this preliminary study, we assessed the effectiveness and safety of bosentan in a case series of 8 adults with TAO and severe ischemic ulceronecrotic lesions who were treated with bosentan after inadequate response to platelet inhibitors, vasodilators, and intravenous alprostadil. Additionally, we reviewed 18 well-documented patients with refractory TAO treated with bosentan, which was previously reported (PubMed 1965–2015). These 26 patients formed the basis of our present analysis. All were current smokers.

The median duration of bosentan treatment (SD) was 4.5 ± 4 months (range 3–16). Eleven patients (42%) were unable to completely abstain from smoking during their follow-up. With bosentan treatment, no new ischemic lesions were observed in the target extremities. A complete therapeutic response was achieved in 80% of patients, whereas a partial response was observed in 12%. Two patients (8%) ultimately required amputation despite treatment.

After discontinuation of bosentan, patients were followed for a median of 20 ± 14 months (range 3–60). Two patients whose trophic lesions had healed relapsed.

When comparing patients who gave up smoking with those who were unable to completely abstain from smoking during follow-up, no significant differences were found in efficacy outcomes. Four patients (15%) developed adverse events, requiring bosentan discontinuation in 1 case.

These preliminary data suggest that bosentan may be considered a therapeutic option for treatment of cases of severe TAO refractory to conventional treatment, and merit further evaluation in larger controlled, randomized clinical studies.

## 1 Introduction

Thromboangiitis obliterans (TAO) or Buerger disease is a distinct form of systemic vasculitis of unknown etiology, though strongly linked to cigarette smoking.^[1,2]^ It affects the small and medium-sized arteries and veins in the upper and lower extremities. Symptoms generally begin with coldness of the fingers and toes, and intermittent claudication, then progress to ischemic pain at rest, and ultimately result in the development of ischemic ulcerations and gangrene that can necessitate amputation.^[1,2]^

The cornerstone of therapy is complete abstinence from tobacco in any form, which is the only way to halt the disease progression and reduce the risk of amputation.^[1,2]^ In addition to the discontinuation of tobacco, very few pharmacological and surgical options of controversial efficacy are available to date. Different treatments such as antiplatelet agents, anticoagulants, and vasodilators (including calcium channel blockers, pentoxyfylline, and cilostazol) are widely used, although there is no proven evidence of their palliative benefits.^[3,4]^ Prostaglandin analogs are beneficial when administered intravenously, although they are no better than placebo on oral administration, and the beneficial effects are transient and disappear after treatment is stopped.^[3]^ Surgical techniques (revascularization, sympathectomy, Ilizavor technique, and omentopexy omentum autografts) are limited in scope and usefulness.^[4]^ New therapeutic options with greater efficacy are clearly needed to properly manage these patients.

Endothelial dysfunction appears to be of relevance in TAO,^[5–7]^ and an increase in the level of endothelin 1 (ET-1)—a potent vasoconstrictor—has been observed in patients with clinically active disease and necrotic lesions.^[8]^ Based on these findings, bosentan—a competitive antagonist of ET-1 at the endothelin-A (ET-A) and endothelin-B (ET-B) receptors—is beginning to be used in severe and refractory cases of TAO, and has effectiveness and a good safety profile.^[9–15]^

In this preliminary study, we assessed the effectiveness and safety of bosentan in a case series of patients with severe TAO refractory to conventional treatment, including platelet inhibitors, vasodilators (calcium channel blocker and/or pentoxyfylline), and intravenous prostaglandins. Current evidence of the therapeutic use of bosentan in this complex situation was also analyzed through a systematic review of the literature.

## 2 Patients and methods

### 2.1 Patient selection

The sample included eight patients with TA recruited at three hospitals in Barcelona (Spain), all fullfiling the diagnostic criteria proposed by Olin et al^[1,2]^ and Shinoya et al^[16]^: onset before the age of 50 years; current (or recent past) tobacco use; distal extremity ischemia (infrapopliteal and/or intrabrachial), such as claudication, rest pain, ischemic ulcers, and gangrene documented with noninvasive testing; exclusion of connective tissue diseases and hypercoagulable states; exclusion of a proximal source of emboli with echocardiography and arteriography; and consistent arteriographic findings in the involved and clinically uninvolved limbs including multiple segmental occlusions of distal arterial sections (distally from the elbow and knee); chronic vascular occlusion due to secondary thrombosis; absence of atherosclerotic lesions, such as calcification of vascular walls; tapering and abrupt obstruction of vessels, a twisting course of the involved vessels; and “corkscrew” or “tree-root” collaterals.

Patients were selected to be treated with bosentan if they met the following inclusion criteria: severe distal extremity ischemia with nonhealing ulceronecrotic lesions present for at least 4 weeks and with inadequate response to conventional treatment, including platelet inhibitors (aspirin or clopidogrel), vasodilators (calcium channel blocker and pentoxyfylline), and intravenous alprostadil (PGE-1) for 21 days; and non-candidates for surgical or endovascular revascularization of the extremity studied.

A clinical pilot, open-label, uncontrolled, prospective study was designed in which these patients received treatment with bosentan in a compassionate use program. Informed consent was obtained from each patient, and their clinical records were anonymized and de-identified before analysis. The study was approved by our institutional ethics committee and was conducted in accordance with the principles of the Declaration of Helsinki and the International Conference for Harmonization.

All patients received bosentan p.o. at a dose of 62.5 mg twice daily during the first month, which was thereafter up-titrated to 125 mg twice daily each month if significant adverse events attributable to bosentan were ruled out. This full-dose regimen (125 mg/12 h) was maintained until complete healing of the distal ischemic trophic lesions was achieved, provided that the liver function tests and blood cell counts remained within the normal ranges. Concomitant medication with platelet inhibitors (aspirin or clopidogrel) and vasodilators (calcium channel blocker and/or pentoxyfylline) was maintained at the discretion of the referring physician. Patients were given analgesic treatment as necessary to control pain at rest. The ischemic lesions and necrotic ulcers were treated with standard daily care and antibiotic therapy as necessary.

Inpatient and outpatient charts were comprehensively reviewed following a specifically designed protocol that addressed smoking status during the study period, efficacy data (evolution of ischemic pain and trophic lesions, presence/absence of major or minor amputation), and safety data (tolerability and side effect profile of bosentan). The endpoint of patient follow-up was the date of the last clinic visit. The study endpoints were clinical improvement rate, the need for amputation (major or minor), and the occurrence of relapses. Complete therapeutic response was defined as the complete healing of the distal ulceronecrotic lesions with disappearance of ischemic pain. Partial response was considered in cases with significant improvement of ulceronecrotic lesions without complete healing.

### 2.2 Literature search strategy and selection criteria

In addition to our case series, we analyzed current evidence on the therapeutic use of bosentan in patients with TAO using a systematic review of reports published in indexed international journals (not reviews, congress abstracts, or unpublished results). Searches were conducted in the PubMed database (ie, including MEDLINE, National Library of Medicine, and PubMed Central) for the period between January 1965 and December 2015 using the strategies recommended by the Cochrane handbook. Search terms included *“*bosentan”, “thromboangiitis obliterans,” and “Buerger disease.” Only English and Spanish-language reports were selected for review. The references of the studies obtained were then examined to identify additional reports.

The MEDLINE search resulted in 9 articles.^[4,9–15,17]^ After evaluation of the full text, 2 of these articles were excluded because they were reviews.^[4,17]^ In addition, we excluded 7 of the 19 cases reported by De Haro et al^[11]^ because their clinical characteristics were not sufficiently detailed to be individually analyzed.

Therefore, 6 articles were finally selected for review, identifying 18 well-documented cases of patients with TAO refractory to conventional treatment who were treated with bosentan.

### 2.3 Statistical analysis

Qualitative variables were described by frequencies and percentages, and quantitative variables were described by mean or median ± standard deviation (SD) and range. Comparison between groups was made using the chi-square test for categorical data or the Fisher exact test when the expected values were less than 5. Statistical significance was defined as *P* ≤ 0.05.

## 3 Results

A total of 8 consecutive patients were included in the study. Their main clinical characteristics and outcome are summarized in Table 1. All patients had previously been treated with a 21-day prostaglandin regimen, platelet inhibitors (aspirin or clopidogrel), and vasodilators (calcium channel blocker and/or pentoxyfylline) without significant clinical improvement in the symptoms or healing of their ischemic ulceronecrotic lesions.

**Table 1:**
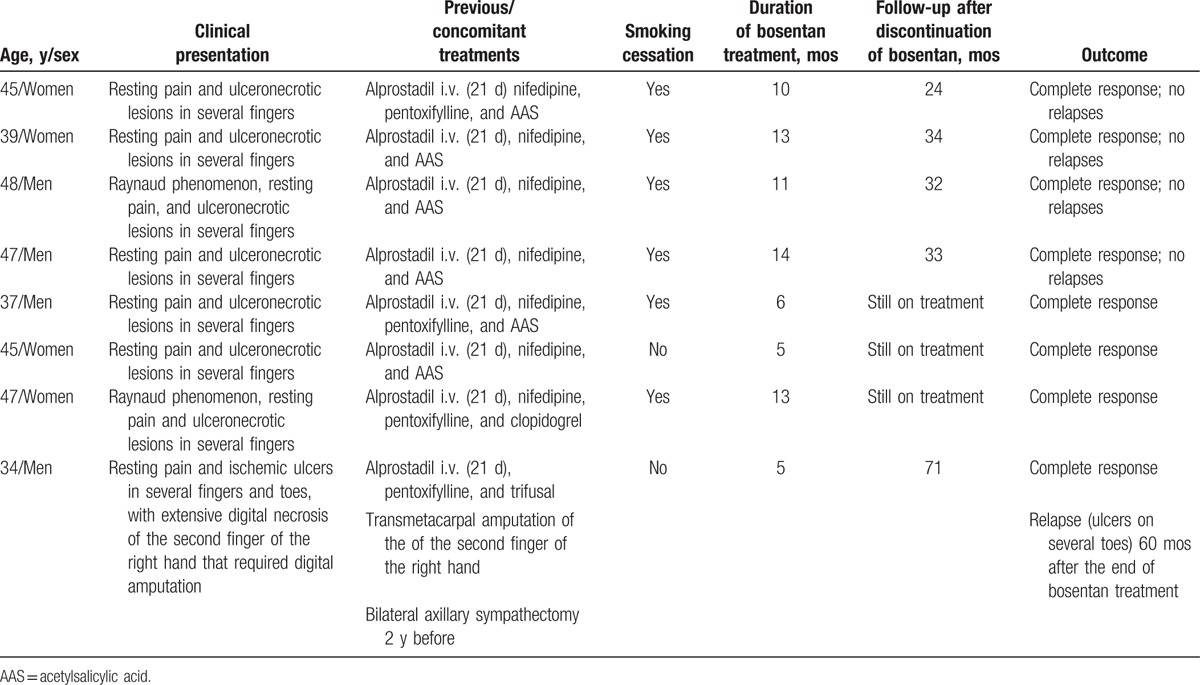
Main clinical characteristics and outcomes of our eight patients.

The main clinical characteristics and outcomes of the 18 patients obtained from the literature are summarized in Table 2. Including the 8 patients presented here, 26 cases of refractory TAO treated with bosentan were available for review. The global analysis of these patients provided the information described under the following subheadings.

**Table 2:**
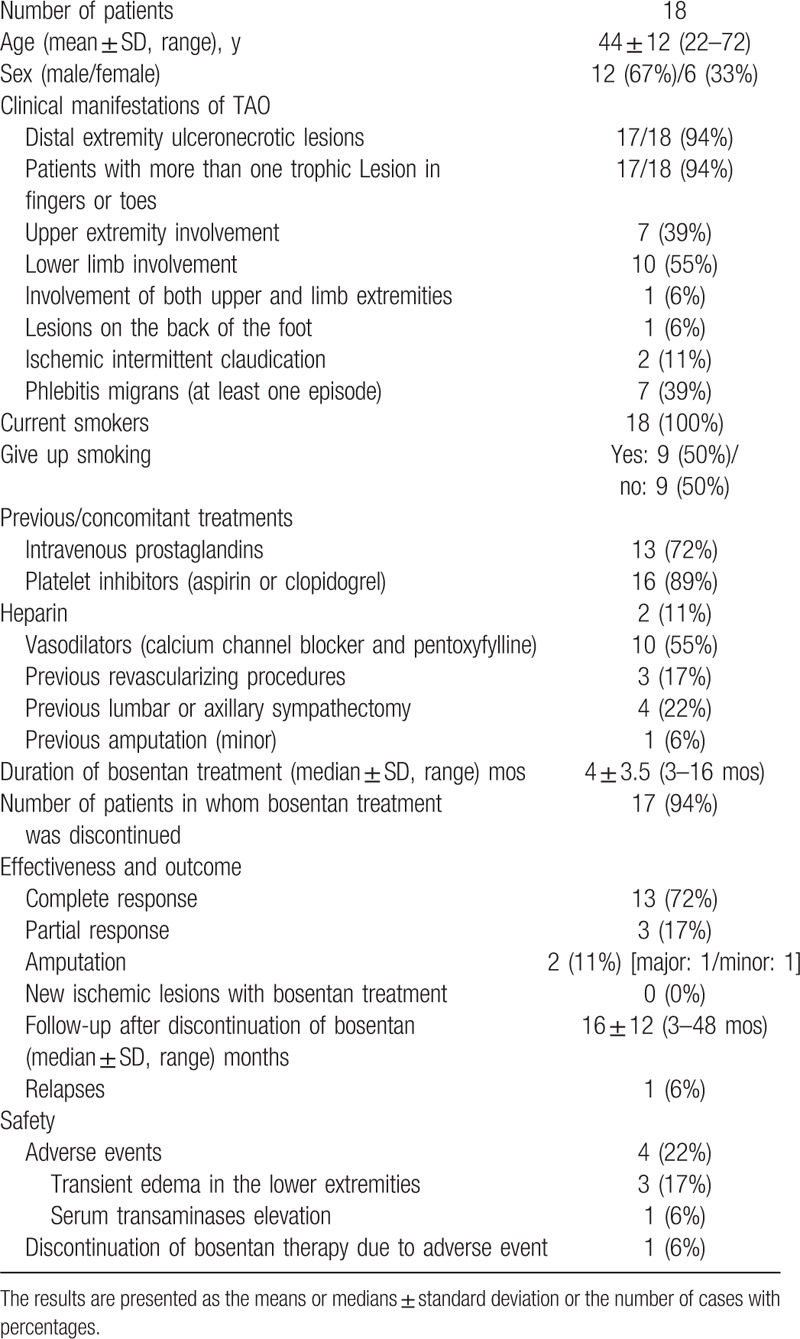
Clinical characteristics and outcomes of 18 reported patients with refractory thromboangiitis obliterans (TAO) treated with bosentan.

### 3.1 Demographic data

Of the 26 patients, 16 were men (61.5%) and 10 (38.5%) were women, with ages ranging from 22 to 72 years (mean 44 ± 11 years). All were current smokers. Two (8%) patients were also frequent cannabis and cocaine abusers.

### 3.2 Clinical manifestations of TAO

All but 1 patient (96%) presented with distal ischemic ulcers and/or digital necrosis, generally in the form of necrotic irregular eschars 1 to 1.5 cm in diameter in the pads of the fingers or toes, or in the periungual areas, and more rarely as an extensive necrosis and toe gangrene. The remaining patient only had ischemic pain at rest in the legs with skin integrity at baseline.

The upper extremities were involved in 50% (13/26) of the patients, the lower limbs were involved in 38% (10/26), and 12% (3/26) presented simultaneous involvement of upper and lower extremities.

Twenty-five patients (96%) had more than 1 ulceronecrotic lesion in the fingers or toes. Only 1 patient (4%) presented with lesions on the back of the foot at baseline.

Seven patients (27%) previously had at least 1 episode of phlebitis migrans, Raynaud phenomenon was present in 2 (8%) patients, and ischemic intermittent claudication was present in another 2 (8%).

### 3.3 Previous and concomitant treatments

Before treatment with bosentan, 81% (21/26) of the patients had previously been treated with intravenous prostaglandins (in all but 1 case with a 21-day regimen). Antiplatelet drugs were used in 92% (24/26) of the patients, heparin in 8% (2/26), and vasodilators (calcium channel blocker and/or pentoxyfylline) in 69% (18/26).

Three (12%) of the 26 patients had undergone revascularizing procedures, 5 (19%) had a previous lumbar or axillary sympathectomy, and 2 (8%) had required previous minor amputation.

Eleven patients (42%) were unable to completely abstain from smoking during the follow-up.

### 3.4 Effectiveness and outcome

The duration of bosentan treatment (median ± SD) was 4.5 ± 4 months (range 3–16). At the time of the last follow-up, 4 out of 26 patients (15%) were still taking bosentan, with a median treatment duration of 9 ± 4 months. In the remaining 22 patients, bosentan therapy was discontinued.

During follow-up with bosentan treatment, no new ischemic lesions were observed in the target extremities. A complete therapeutic response was achieved in 80% (21/26) of the patients, whereas partial response (significant improvement of ulceronecrotic lesions without complete healing) was observed in 12% (3/26). Two patients (8%) ultimately required amputation despite treatment (major in 1 case and minor in the other).

In some of these patients, the improvement in distal circulation was confirmed by objective methods such as digital arteriography with subtraction, angio-magnetic resonance imaging (MRI) or angio-TC (12 patients), brachial artery flow-mediated dilation test (12 patients), nailfold capillaroscopy (2 patients), or digital plethysmograph (4 patients). In all patients monitored with the brachial artery flow-mediated dilation test (BAFMD), bosentan therapy significantly improved their baseline values (mean: 1.8 at baseline, 6.6 at the end of the bosentan treatment, and 12.7 at 3 months after the end of the treatment; *P* < 0.01).

After discontinuation of bosentan, patients were followed for a median of 20 ± 14 months (range 3–60). Two patients whose trophic lesions had healed relapsed 3 and 60 months after the end of bosentan therapy. In 1 of these cases, a new 4-month course of bosentan was sufficient to heal the lesions.

When comparing patients who gave up smoking with those who were unable to completely abstain from smoking during follow-up, no significant differences were found in the outcome measures (smoking vs no smoking): complete response 73% versus 87% (*P* = 0.373); partial response 18% versus 7% (*P* = 0.364); amputation (major or minor) 9% versus 7% (*P* = 0.819); and relapses 18% versus 0% (*P* = 0.086).

### 3.5 Safety

Four patients (15%) developed adverse events due to bosentan therapy: 1 case of serum transaminase elevation leading to withdrawal of treatment at 3 months and 3 cases of transient edema in the lower extremities, consistent with the vasodilatory effect of the distal bed associated with bosentan use.

## 4 Discussion

Despite not being life-threatening, TAO has a poor functional prognosis, often requiring partial or total surgical limb amputations. In addition to discontinuation of cigarette smoking, only intravenous prostaglandin has demonstrated proven efficacy, but its effect fades upon finishing treatment (21–28 days).^[3]^ More recently, bosentan has been introduced in refractory cases of TAO, bringing in a new alternative for prolonged oral treatment.^[9–15]^

The current evidence for the effectiveness of bosentan in TAO is based solely on several case reports and 2 small pilot observational studies, including ours. Although it is not yet possible to make definite recommendations, the global analysis of these preliminary data supports the off-label use of bosentan as a second-line therapy in patients with severe refractory TAO. A complete therapeutic response was achieved in 80% of these patients, with an amputation rate (major and minor) of only 8%. It should be highlighted that this excellent clinical response was achieved, despite the fact that 42% of the patients continued to smoke. These results compare favorably with data from previous studies, which reported an amputation rate of 19% to 65% among the patients who continued to smoke.^[18,19]^

Of interest, the clinical improvement was accompanied by a significant improvement in distal circulation confirmed by objective methods such as imaging techniques (digital arteriography, angio-MRI, or angio-TC),^[11–14]^ BAFMD,^[11,12]^ nailfold capillaroscopy,^[14]^ or digital plethysmograph.^[14]^ Long-term follow-up confirmed the sustained clinical response with a low rate of relapses (8%) after the end of bosentan therapy. Although the response rates were numerically better among patients who gave up smoking, there were no significant differences in the efficacy outcomes when compared with patients who were unable to completely abstain from smoking during follow-up. The safety profile of bosentan therapy was favorable, although ongoing vigilance for the known adverse reactions is required.

The mechanism through which bosentan acts on TAO is unclear. One of the key findings in the immunopathogenesis of this disease is the injury to vascular endothelium, resulting in activation of the inflammatory response.^[4,20,21]^ Pathohistologically, TAO lesions are categorized into 3 phases, including acute, subacute, and chronic, according to the thrombus pattern and the nature of the inflammatory cells.^[20]^ Signs of endothelial activation and proliferation, and also the presence of immunocompetent cells, are only seen in acute-type lesions.^[20]^ Endothelial cells play a key role in initiation and perpetuation of the inflammatory response. Immunoglobulin and complement deposition, and also CD4+ and CD8+ T lymphocytes, CD20+ B lymphocytes, and S100 positive dendritic cells are found alongside the lamina elastica interna, which become structurally altered, but are typically preserved.^[4,20,21]^ An impaired endothelium-dependent vasorelaxation in the peripheral vasculature, even in the nondiseased limbs, has been observed in TAO.^[5–7]^ Bosentan has been shown to improve endothelial function in patients with systemic sclerosis,^[22]^ and, according to the results of the study of De Haro et al,^[11,12]^ who measured it indirectly by means of BAFMD, also in TAO patients. Current evidence points to a greater, or faster, effect of bosentan on the endothelium compared with its effect as an antifibrotic.^[23]^ Various types of autoantibodies have been identified in this condition, including antiendothelial antibodies and agonistic autoantibodies directed against ET-A-receptor loop 1, potentially promoting vasospasm, compromising microcirculation, damaging vessel structures, and inducing proliferative processes.^[24,25]^ In addition, an elevated serum ET-1 level has been observed in patients with clinically active disease and necrotic lesions, supporting a possible mechanistic explanation of the clinical benefit of bosentan in TAO.^[8]^ ET-1 is a potent endogenous vasoconstrictor that is mainly secreted by endothelial cells. It acts through 2 types of receptors: ET-A and ET-B. Bosentan acts as a competitive antagonist of ET-1 at the ET-A and ET-B receptors. Apart from vasoconstrictive actions, ET-1 causes fibrosis of vascular cells and stimulates production of reactive oxygen species.^[26,27]^ It is claimed that ET-1 induces proinflammatory mechanisms, increasing superoxide anion production and cytokine secretion.^[26,27]^ A recent study has shown that ET-1 is involved in the activation of transcription factors such as NF-κB and the expression of proinflammatory cytokines such as tumor necrosis factor (TNF)-α, interleukin (IL)-1, and IL-6.^[26,27]^

When interpreting the results of our study, one needs to consider the potential limitations derived from its observational nature, the small sample size, and the lack of multiple-center data. In addition, we cannot ignore the pitfalls inherent in any systematic review, including the relatively small number of identified patients, the retrospective design, and incomplete follow-up data in some cases. In addition, the high rate of efficacy observed may be partially explained by the fact that most reports include cases with a favorable response, whereas cases without such a response are often not reported.

In summary, our results and those previously reported support that bosentan may be considered a therapeutic option for treatment of cases of severe TAO refractory to conventional treatment. We would like to highlight the excellent clinical response despite the fact that nearly half of patients continued to smoke. Further randomized clinical studies with large sample sizes are required to confirm these preliminary open-label data and establish the length of therapy and the appropriate use of concomitant medication.
